# Exposure to parasites increases promiscuity in a freshwater snail

**DOI:** 10.1098/rsbl.2013.1091

**Published:** 2014-04

**Authors:** D. M. Soper, K. C. King, D. Vergara, C. M. Lively

**Affiliations:** Department of Biology, Indiana University, Bloomington, IN, USA

**Keywords:** parasite exposure, Red Queen hypothesis, mating behaviour, genetic variation, multiple mating

## Abstract

Under the Red Queen hypothesis, outcrossing can produce genetically variable progeny, which may be more resistant, on average, to locally adapted parasites. Mating with multiple partners may enhance this resistance by further increasing the genetic variation among offspring. We exposed *Potamopyrgus antipodarum* to the eggs of a sterilizing, trematode parasite and tested whether this altered mating behaviour. We found that exposure to parasites increased the number of snail mating pairs and the total number of different mating partners for both males and females. Thus, our results suggest that, in host populations under parasite-mediated selection, exposure to infective propagules increases the rate of mating and the number of mates.

## 1. Introduction

Infectious diseases are ubiquitous and often substantially reduce host fitness [1]. According to the Red Queen hypothesis, selection imposed by virulent, coevolving parasites can select for sexual reproduction over asexual reproduction, because of the diversifying genetic effects that recombination and outcrossing have on offspring [2–4]. The genetic diversity of offspring may be further increased if females choose dissimilar mates [5–7] or mate with multiple males [8–10]. Multiple mating, in particular, has been shown to generate higher genotypic diversity among offspring than sex and recombination alone [11]. Here, we test the hypothesis that exposure to parasites increases multiple mating in a freshwater snail.

We exposed the New Zealand freshwater snail, *Potamopyrgus antipodarum*, to the infective eggs of its sterilizing, trematode parasite *Microphallus* sp. ‘livelyi’ [12]. Previous studies on this host–parasite system have revealed parasite-mediated selection against common host genotypes, as well as strong local adaptation by the parasite [13,14]. We have also found direct evidence for multiple paternity in natural populations of *P. antipodarum* [15]. The goal for this study was to determine whether sexual females of this snail could be induced by exposure to parasites to increase their rate of mating and the number of partners.

## 2. Material and methods

*Potamopyrgus antipodarum* is a freshwater snail commonly found in lakes and streams throughout New Zealand. Individual snails are either triploid parthenogenetic females or diploid dioecious sexuals [16]. Populations can be mixed, having both clonal and sexual individuals, or they can comprise only of clonal individuals [17,18].

This snail species is the first intermediate host for several species of digenetic trematodes, of which *Microphallus* sp. ‘livelyi’ is the most common parasite in lake populations [12,19,20]. This parasite produces encysted larvae (i.e. metacercariae) in the snail host after about three months under laboratory conditions, and the snails are sterilized from infection. The parasite develops into a hermaphroditic adult stage after ingestion by the definitive host (ducks and wading birds) and produces eggs within several days. These eggs are then passed into the environment with faeces of infected birds. Previous studies have shown that the snails can become infected after exposing them to *Microphallus* eggs collected from the faeces of ducks and wading birds [21,22].

We examined whether exposure to field-collected *Microphallus* eggs increased the number of mating pairs formed and the number of mating partners per individual *P. antipodarum*. We isolated male and female snails from an outbred, sexual lineage that was descended from snails originally collected from Lake Alexandrina (South Island, New Zealand) and then maintained in the laboratory for over five years. For each of 24 experimental units, 17 male and 17 female snails larger than 2.5 mm (adult size) were individually painted with a unique colour of nail polish, and then placed in a container with 1 l water. One of four possible duck-faeces inocula were then added to each experimental unit, with six replicates per treatment: (i) 1 ml duck faeces (collected from Lake Alexandrina; containing approximately 616 *Microphallus* eggs/snail and naturally occurring bacteria (‘natural’ treatment), (ii) 1 ml autoclaved (sterilized) duck faeces (‘sterile’ treatment), (iii) 1 ml autoclaved duck faeces to which bacteria-laden (but trematode-free) water from the duck faeces was added after autoclaving (‘bacteria’ treatment), (iv) 1 ml of ‘bleached’ duck faeces prepared using a ‘bleaching protocol’ that removes bacteria, but does not kill worm eggs (‘parasite’ treatment) [23]. Treatments (ii)–(iv) were designed to control for the possibility that snails were responding to exposure to bacteria and/or to duck faeces *per se*, rather than to *Microphallus*.

Twenty-four hours following exposure, we counted the number of mating pairs and recorded the identity of each mating individual in each replicate container three times per day (10.00, 13.00 and 16.00) over each of 10 days.

### (a) Statistical analysis

IBM SPSS Statistics v. 20.0 (NY, USA) was used for all analyses. We used linear mixed models to examine how the treatments affected the number of mating pairs formed and the number of mating partners per female and per male. ‘Parasites’ and ‘bacteria’ were analysed as separate crossed fixed factors. Day of exposure was a separate fixed factor, and replicate was a random factor. Owing to the large sample sizes used in the experiment, our tests were robust to the moderately skewed distribution of the residuals [24]. Parameters were estimated using restricted maximum likelihood, and the significance of the fixed factors was determined using type III *F*-tests. We used Dunnett's *post hoc* tests to determine whether the number of mating pairs formed and the number of partners in exposure treatments differed from control treatments.

## 3. Results

**Figure 1.**
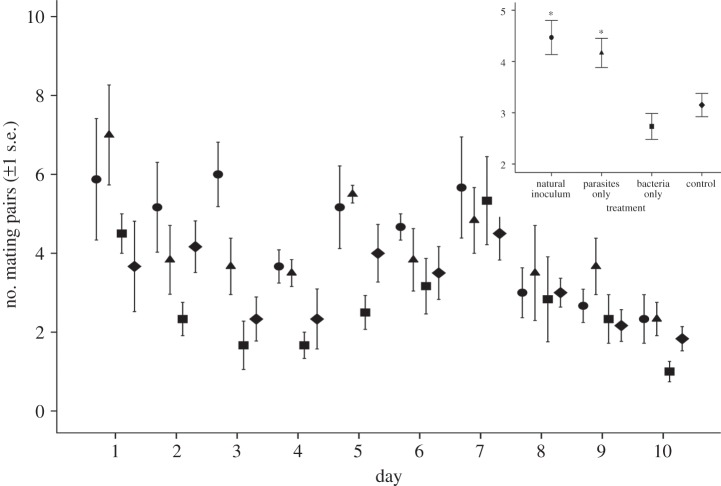
Number of mating pairs observed during parasite exposure (±1 s.e.). Number of mating pairs counted per day over the 10 days of exposure in the control (diamonds) and treatments: natural inoculum (circles), parasite inoculum (triangles) and bacteria inoculum (squares). The inset figure shows the mean number of mating pairs averaged across the 10-day exposure period. Asterisks indicate significant *p*-values (*p* < 0.05) for *post hoc* Dunnett's tests comparing exposure treatments with the control.

**Figure 2.**
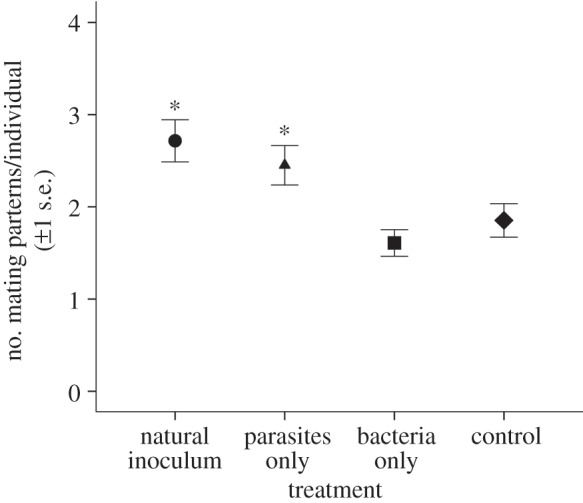
Mean number (±1 s.e.) of mating partners per female in the control (sterile) and three exposure treatments. Asterisks indicate significant *p*-values (*p* < 0.05) for post hoc Dunnett's tests comparing exposure treatments with the control.

Exposure to parasites increased the number of mating pairs formed and the number of different partners in those pairs (table 1, and figures 1 and 2). The effect on mating pairs was only present in the two exposure treatments with parasites, and not in the bacterial treatment or control (figure 1 inset; Dunnett's test: natural *p* < 0.001, parasite *p* = 0.013). Day of exposure also affected the number of mating pairs observed among treatments (table 1), with the most mating pairs counted on the first day (figure 1). While all male snails mated at least once, some females were never observed in a mating pair. When compared with the control, higher numbers of mating partners per female were detected in both the parasite treatments (Dunnett's test: natural *p* = 0.003, parasite-only *p* = 0.036). Similarly, there were significantly more mating partners per male found in the natural duck faeces treatment (*p* = 0.017) relative to the control, but only marginally more partners in the parasite-only treatment (*p* = 0.107). Finally, no difference was detected in the number of female mating partners between the bacteria-only treatment and the control (*p* = 0.977).

**Table 1.** Linear mixed models of the effect of parasite and bacterial exposure on the number of snail mating pairs and the number of male partners/female.

	no. mating pairs			no. male partners/female		
	d.f.	*F*	*p*	d.f.	*F*	*p*
intercept	1, 5	784.816	<0.001	1, 404	490.563	<0.001
parasites	1, 224	38.944	<0.001	1, 404	19.179	<0.001
bacteria	1, 224	0.152	0.945	1, 404	0.003	0.960
parasites × bacteria	1, 224	2.093	0.072	1, 404	1.713	0.191
day	9, 224	7.959	<0.001

## 4. Discussion

Under the Red Queen Hypothesis, outcrossing can produce genetically variable progeny, which may be more resistant than asexually produced progeny, on average, to coevolving parasites [2–4,13,25]. Moreover, the number and choice of mates in the population may enhance this resistance by further increasing genetic variation among offspring [23]. In natural populations of *P. antipodarum* with high frequencies of infection, the snails produce broods with multiple sires [15], and here, we show that parasite exposure alone can increase the mating rate and number of different mating partners, which could account for multiple paternity. Taken together, these results suggest that parasite-mediated selection can favour the diversification of broods via multiple paternity.

Presently, we can only speculate on the underlying mechanisms of these behavioural changes following parasite exposure. In other systems, host behaviour is altered by parasite-secreted chemicals, parasite-mediated manipulation of the central nervous system or encystation within neurological or muscular tissues [26]. However, these behavioural modifications have typically been observed when infections have developed. We found that exposure, not necessarily infection (which takes at least three months to develop to a transmissible stage), affects the short-term sexual behaviour of *P. antipodarum*. The increase in polyandry that we observed may act to increase the number of sires (and thus genetic diversity) within the brood, but may additionally increase the genetic quality of offspring by generating sperm competition and an opportunity for cryptic female choice [27]. In this way, increased polyandry in response to parasite exposure may directly increase offspring survival [28] if the offspring are less likely to become infected.

In summary, we found that exposure to parasites drives up the number of mating partners and mating pairs formed in snail populations. These results are consistent with previous studies suggesting that natural populations of this snail are under parasite-mediated selection for sexual reproduction, favouring high genetic diversity [13,21]. Beyond sex and recombination, multiple mating could further increase genetic diversity for resistance among offspring. Thus, the changes in sexual behaviour we observed might be important for countering the constant risk of infection by parasites in nature. Future studies may benefit from investigating how other environmental factors influence mating behaviour and the genetic diversity of offspring production after parasite exposure.

## Data accessibility

Data available from the Dryad Digital Repository: doi:10.5061/dryad.9c6q0.
